# V-Raf murine sarcoma viral oncogene homolog B1 (BRAF) as a prognostic biomarker of poor outcomes in esophageal cancer patients

**DOI:** 10.1186/s12876-021-01671-2

**Published:** 2021-02-23

**Authors:** Aihemaijiang Kuerbanjiang, Maimaitiyiming Maimaituerxun, Yanjun Zhang, Yiliang Li, Gang Cui, Aibaidula Abuduhabaier, Abuduwaili Aierken, Buya Miranbieke, Meilikezati Anzaer, Yusufu Maimaiti

**Affiliations:** 1 Department of Gastroenterology, People’s Hospital of Xinjiang Uygur Autonomous Region, Urumqi, 830000 China; 2 Department of General Surgery, People’s Hospital of Xinjiang Uygur Autonomous Region, Urumqi, 830000 China; 3 Department of Clinical Research Center, People’s Hospital of Xinjiang Uygur Autonomous Region, Urumqi, 830000 China

**Keywords:** V-Raf murine sarcoma viral oncogene homolog B1, BRAF, Esophageal cancer, Biomarker, Prognosis

## Abstract

**Background:**

Esophageal cancer is one of the most aggressive malignancies, and is associated with multiple genetic mutations. At present, the v-Raf murine sarcoma viral oncogene homolog B1 (BRAF) gene mutation has been observed in esophageal cancer and is associated with poor prognosis. This study aimed to investigate the protein expression of BRAF in esophageal cancer and determine its effect on patient outcomes.

**Methods:**

We used immunohistochemistry to detect the expression of BRAF via tissue microarrays in esophageal cancer samples, the Kaplan–Meier method to perform survival analysis, and the Cox proportional hazards regression model to explore the risk factors of esophageal cancer. The role of BRAF in the proliferation, invasion, and metastasis of esophageal cancer was studied by clone formation, scratch test, Transwell invasion and migration test. The tumor-bearing model of BRAF inhibitor was established using TE-1 cells, and corresponding negative control was set up to observe the growth rate of the two models.

**Results:**

The results revealed that BRAF overexpression was significantly correlated with Ki67 (*P* < 0.05). Survival analysis showed that BRAF overexpression contributed to a shorter overall survival (*P* = 0.014) in patients with esophageal cancer. Univariate and multivariate regression analyses demonstrated that BRAF was a prognostic factor for poor esophageal cancer outcomes (*P* < 0.05). Small interfering RNA knockdown of BRAF significantly reduced the cell clone formation rate compared to the control group. Transwell assay analysis showed that the migration and invasion of cells in the experimental group were significantly inhibited relative to the control group, and the inhibition rates of the small interfering RNA group were 67% and 60%, respectively. In the scratch test, the wound healing ability of the BRAF knockdown group was significantly weaker than that of the control group. There were significant differences in tumor growth volume and weight between the two groups in nude mice.

**Conclusion:**

BRAF overexpression may serve as an effective predictive factor for poor prognosis.

## Background

Esophageal cancer (EC) is the eighth most common cancer and the sixth most common cause of death globally [1]. EC remains a significant health problem in the world because the 5-year survival rate is low. Worldwide statistics have revealed that the incidence of EC increases with age, and men are more likely to be diagnosed with the disease than women at a ratio of 3:1 [2]. Many EC patients are already locally advanced or advanced at the time of diagnosis, and EC is susceptible to recurrence and metastasis, highlighting the main reasons for poor EC prognosis. Although there are many factors that influence the prognosis of EC, additional influential factors need to be studied further. A new index is necessary to enable more accurate prognostic predictions and provide the best preoperative counseling to patients [3]. The coding products of v-Raf murine sarcoma viral oncogene homolog B1 (BRAF) occur through the epidermal growth factor receptor (EGFR) signal transduction network, which plays a crucial role in multiple tumorigenic processes, including cell cycle progression, angiogenesis, metastasis, and protection of the cancer cell from apoptosis [4, 5]. Mutations in the Kirsten ras 1 (KRAS) and BRAF genes may be predictive of drug responses that are directly linked to the EGFR pathway [6].

In this study, we detected BRAF protein expression in EC tissues, studied the effect of BRAF on the invasion and metastasis of TE-1 (human esophageal cancer cell) cells in vitro, and explored the value of the BRAF gene in early EC diagnosis as well as the possibility of predicting prognosis to a provide theoretical basis for using BRAF inhibitors as targeted drugs in EC treatment.

## Methods

### Patients and samples

This study was approved by the Human Research Ethics Committee of the People’s Hospital of Xinjiang Uygur Autonomous Region. We purchased 180 EC tissue microarrays (TMAs, No. HEsoS180Su05-M-141) from Shanghai Outdo Biotech Co., Ltd. (Shanghai, China) for the study, including 105 cancer tissues and 75 adjacent normal tissues. We primarily studied the 105 cancer tissues. All patients underwent precise surgery in accordance with their clinical examinations. All patients were pathologically diagnosed as squamous cell carcinoma and did not receive any treatment before surgery.

Clinical characteristics of the 105 patients, such as gender, age, pathological type, pathological grading, tumor infiltration (T stage), lymph node metastasis (N stage), histological type and AJCC clinical stage, were obtained from their medical records (Table 1). Metastasis status was expressed using the pathological stage of the disease according to the seventh edition of the American Joint Committee on Cancer (AJCC)/International Union Against Cancer tumor, node, metastasis (TNM) classification system.

**Table 1:** Clinicopathological characteristics of esophageal cancer patients

Risk factors	Number of patient	%
Total	105	
Age		
< 60	33	32.1
≥ 60	72	67.9
Gender		
Male	77	73.3
Female	28	26.7
Histological type		
Squamous cell carcinoma	105	100
Pathological type		
Protuberant type	1	1.0
Ulcerative type	62	59.0
Medullary type	36	34.3
Mushroom type	6	5.7
T stage		
T1	6	5.7
T2	12	11.4
T3	84	80.0
T4	3	2.9
N stage		
N0	47	44.8
N1	32	30.5
N2	22	21.0
N3	4	3.8
AJCC clinical stage		
Stage I	5	4.8
Stage II	47	44.8
Stage III	53	50.5
Pathological grading		
I	29	27.6
II	67	63.8
III	9	8.6
Ki67 expression		
< 14%	22	20.9
≥ 14%	83	79.1
p53 expression		
Strongly	67	63.8
Weakly	38	36.2
BRAF expression		
Strongly	87	82.9
Weakly	18	17.1

### Follow-up study

Follow-up evaluations were performed according to the standard follow-up system of the hospital every 6 months after patients were discharged. The deadline for follow-up evaluations was December 31, 2016. All patients were followed up for 0–107 months (average: 31.70 months). The survival period was measured from the date of admission to the date of death or the date of the follow-up deadline.

### siRNAs and transfections

For expression vector, cells were seeded in 6-well plates (2.5 105 cells/35-mm well) and transfected after 18–20 h with the appropriate construct using LipofectAMINE (Invitrogen), according to manufacturer’s protocols. After 24 h, cells were treated as stated in figure legends and harvested. For siRNA silencing, cells were seeded at 2.5 105 cells/35-mm well the day before transfection. Cells were transfected using LipofectAMINE in 1 mL of OPTIMEM with 100 nM BRAF-specific (50-CAGUCUACAAGGGAAAGUG-30), or Silencer™ negative control#1 siRNA (Gene Pharma.). After 6 h incubation with the RNA-complex, medium was replaced and 2 mL of fresh medium containing 10% FBS was added. Cells were treated and harvested at the indicated times after the transfection as stated in figure legends.

### Colony formation assay

TE-1 cells stably expressing BRAF were grown until > 90% confluence in a 10 cm culture dish. The cells were detached and resuspended by trypsinization. We carefully counted the number of cells and diluted them such that 200 cells per dish were seeded into petri dishes. Next, the cells were incubated in a 5% CO_2_ environment at 37 °C. The experiment was repeated three times. After incubation, the media was removed, and crystal violet, formaldehyde, and a methanol fixing/staining solution were added to cover the dish. The cells were stained at room temperature for 20 min and the number of cell clones was calculated.

### Cell migration and Transwell assay

For the cell migration assay, cells in the logarithmic growth phase were seeded into the upper transwell chamber insert (Corning Inc., Corning, NY, USA) at a density of 2 × 10^4^ cells per well. The chamber was placed in a 24‐well plate, in which the upper chamber contained serum‐free cell culture medium and the lower chamber contained 10% fetal bovine serum (FBS). The culture was continued for 24 h. The medium was discarded, and the cells were stained with a crystal violet solution to observe the number of migrated cells. For the cell invasion assay, Matrigel (Corning Inc.) was uniformly applied to the upper chamber prior to cell inoculation. The other procedures matched those of the cell migration experiment.

### Wound healing assay

For the wound healing assay, the cells treated under the aforementioned conditions were seeded and grown to confluence in petri dishes. The cell monolayers were scratched with a 10 μL pipette tip and cultured in 2% FBS. Photographs were taken after 0, 12, and 24 h.

### Tumor model

Four-week-old female BALB/c nude mice were purchased from Beijing Vital River Laboratory Animal Technology Co. Ltd. (Beijing, China) and randomly divided into tumor and negative control groups. The right flank of each nude mouse was subcutaneously injected with 1*10^6^ TE-1 cell, and 7 nude mice in each experimental group were used as experimental subjects. The experimental group was injection BRAF inhibitors (GC12482) every 3 days by caudal vein. Tumor volume was measured every 3 days with Vernier calipers (V = 0.5 × L × W). The mice were killed 30 days after subcutaneous injection. All transplanted tumors were removed from the mice and weighed immediately. The data were collected and input into GraphPad software (GraphPad Software, San Diego, CA, USA) to draw the tumor growth curves.

### IHC and scoring

**Fig. 1:**
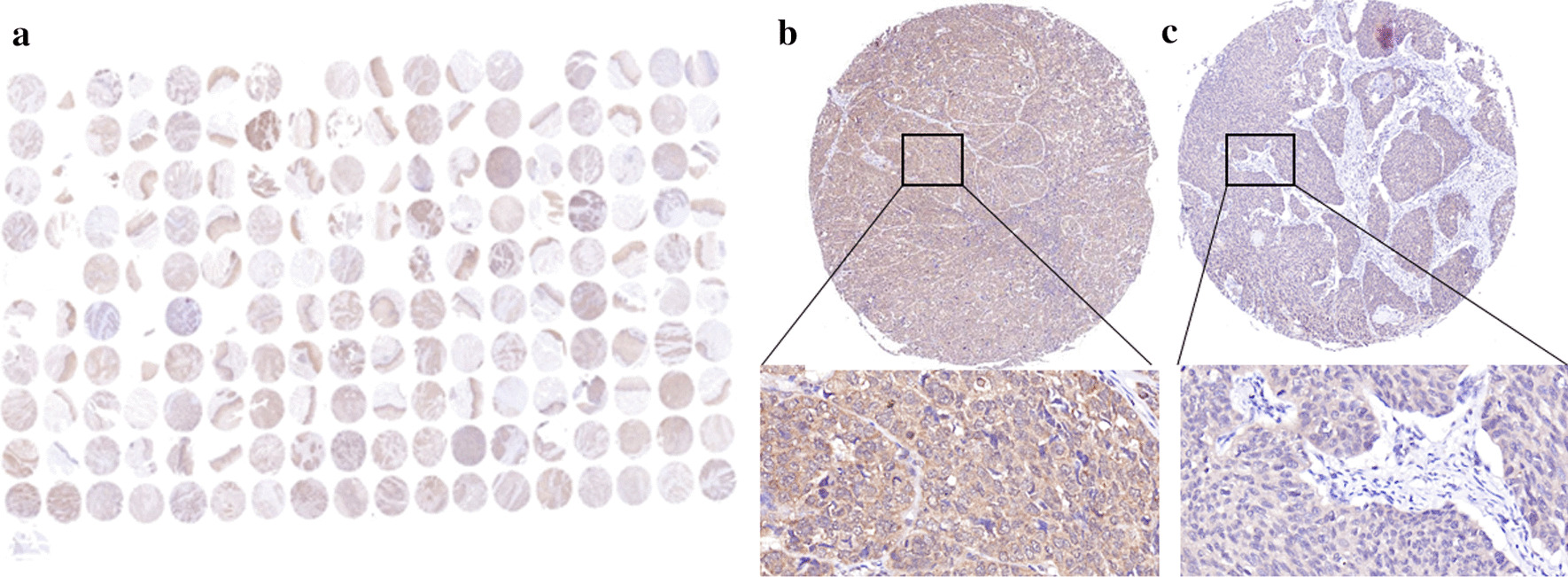
Tissue microarray and immunohistochemistry staining of BRAF in esophageal cancer tissues. **a** Tissue microarray (105 cases of EC tissue and 75 cases of adjacent normal tissue;1 × magnification. **b** Strongly positive expression of BRAF in cancer tissues; 200 × magnification. **c** Weakly positive expression of BRAF in cancer tissues; 200 × magnification

Immunohistochemistry (IHC) staining was used to detect BRAF expression in the EC and adjacent normal tissues TMA (Fig. 1a). To evaluate BRAF expression, we used a BRAF-specific antibody from Abcam (ab68215; Cambridge, UK) at a dilution of 1:100, based on the manufacturer’s protocol. Two experienced pathologists who were blinded to the patients’ clinical information independently evaluated and recorded the IHC results. BRAF-positive cells were judged according to the presence of brown or brown yellow granules in the cells, and the results were calculated by the semiquantitative method. The positive rate of cell staining was 0–5% with 0 points, > 5–30% with 1 point, > 30–60% with 2 points, > 60–100% with 3 points; the intensity of staining was negative with 0 points, weakly positive with 1 point, positive with 2 points, and strongly positive with 3 points. The product of the score of positive cell staining rate and staining intensity was calculated, with 1–4 being weakly positive, 5–8 being moderately positive, and 9–12 being strongly positive. Ki67 high expression was defined as Ki67 staining ≥ 14% of nuclei, and Ki67 low expression was defined as Ki67 staining < 14% of nuclei.

### Statistical analysis

The relationship between BRAF expression and the clinicopathological characteristics of EC patients was assessed using the chi-square test. The Kaplan–Meier method with the log-rank test was used to perform survival analysis, and the Cox proportional hazards regression model was applied to explore the potential prognostic factors for overall survival (OS). All statistical tests were two-sided, and *P* < 0.05 was regarded as statistically significant. All calculations were performed using SPSS Statistics 22.0 software (IBM SPSS, Armonk, NY, USA).

## Results

### Clinicopathological characteristics of EC patients

The detailed clinicopathological characteristics of the 105 patients in this study are provided in Table 1. Approximately two-thirds of the patients were > 60 years old. The male-to-female ratio was 2.75:1. Pathological types included protuberant in 1 case, ulcerative in 62 cases, medullary in 36 cases, and mushroom in 6 cases. Tumor infiltration was at T1 in 8 cases, T2 in 7 cases, and T3 in 64 cases, and T4 in 20 cases. Lymph node metastasis was at N0 in 47 cases, N1 in 32 cases, N2 in 22 cases, and N3 in 4 cases. Regarding pathological grade (I-IV), there were 29 cases of grade I, 67 cases of grade II, and 9 cases of grade III. According to the seventh edition of the AJCC clinical staging system, 5 cases were classified as stage I, 47 were stage II, 53 were stage III.

### Associations between BRAF expression and the clinicopathological features of EC

In our study, EC tumor tissue samples 82.9% (87/105) showed strongly positive BRAF expression, adjacent normal tissues10.7% (8/75) showed strongly positive expression, indicating that BRAF was expressed at a high frequency in EC tissues. We investigated the relationship between BRAF expression levels in EC tissues and the clinicopathological features of EC (Table 2). No significant correlations were found between BRAF expression in EC tissues and gender, age, pathological type, pathological grade, T stage, N stage, AJCC clinical stage, and p53 expression (all *P* > 0.05; Table 2). However, BRAF was correlated with Ki67 (*P* = 0.001; Table 2).

**Table 2:** Relationships between BRAF expression and clinicopathological characteristics

Risk factors	BRAF strongly positive (n = 87)	BRAF weakly positive (n = 18)	χ^2^	*P* value
Age			0.561	0.454
< 60	26 (29.8%)	7 (38.8%)		
≥ 60	61 (70.1%)	11 (61.1%)		
Gender			2.688	0.101
Male	61 (70.1%)	16 (88.8%)		
Female	26 (29.8%)	2 (11.1%)		
Pathological type			6.25	0.100
Protuberant type	0	1 (5.5%)		
Ulcerative type	52 (59.7%)	10 (55.5%)		
Medullary type	29 (33.3%)	7 (38.8%)		
Mushroom type	6 (6.8%)	0		
T stage			0.570	0.903
T1	5 (5.7%)	1 (5.5%)		
T2	10 (11.4%)	2 (11.4%)		
T3	70 (88.4%)	14 (77.7%)		
T4	2 (2.2%)	1 (5.5%)		
N stage			4.809	0.186
N0	38 (43.7%)	9 (50.0%)		
N1	24 (27.6%)	8 (44.4%)		
N2	21 (24.1%)	1 (5.6%)		
N3	4 (4.6%)	0		
AJCC clinical staging			1.756	0.416
Stage I	5 (5.7%)	0		
Stage II	37 (42.5%)	10 (55.5%)		
Stage III	45 (51.7%)	8 (44.4%)		
Pathological grading			0.259	0.879
I	24 (27.5%)	5 (27.7%)		
II	55 (63.2%)	12 (66.6%)		
III	8 (9.1%)	1 (5.5%)		
Ki67			11.067	0.001
< 14%	13 (14.9%)	9 (50.0%)		
≥ 14%	74 (84.1%)	9 (50.0%)		
p53 expression			1.726	0.189
Strongly	59 (67.8%)	15 (83.3%)		
Weakly	28 (31.1%)	3 (16.6%)		

### BRAF expression associations with poor outcomes

**Fig. 2:**
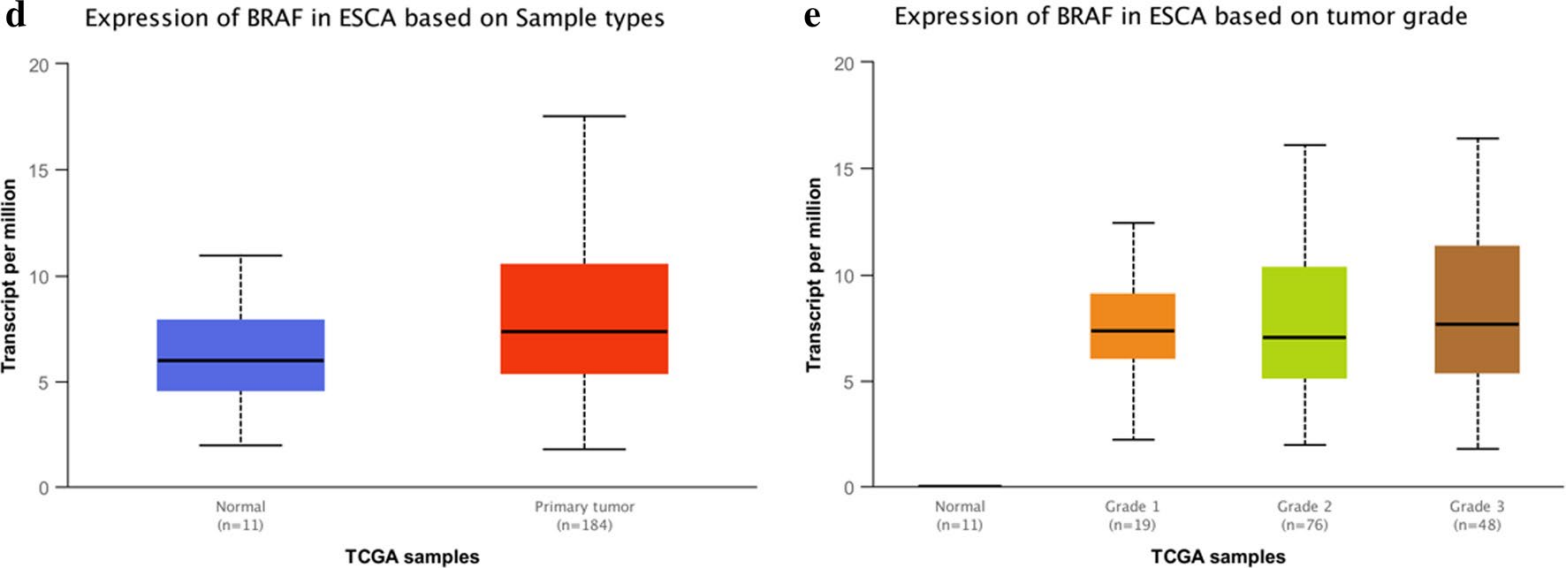
BRAF was overexpressed in EC tissue (**a**). Analysis of BRAF levels in EC tissues compared to those in normal samples and at different disease stages, using TCGA databases (**b**)

**Fig. 3:**
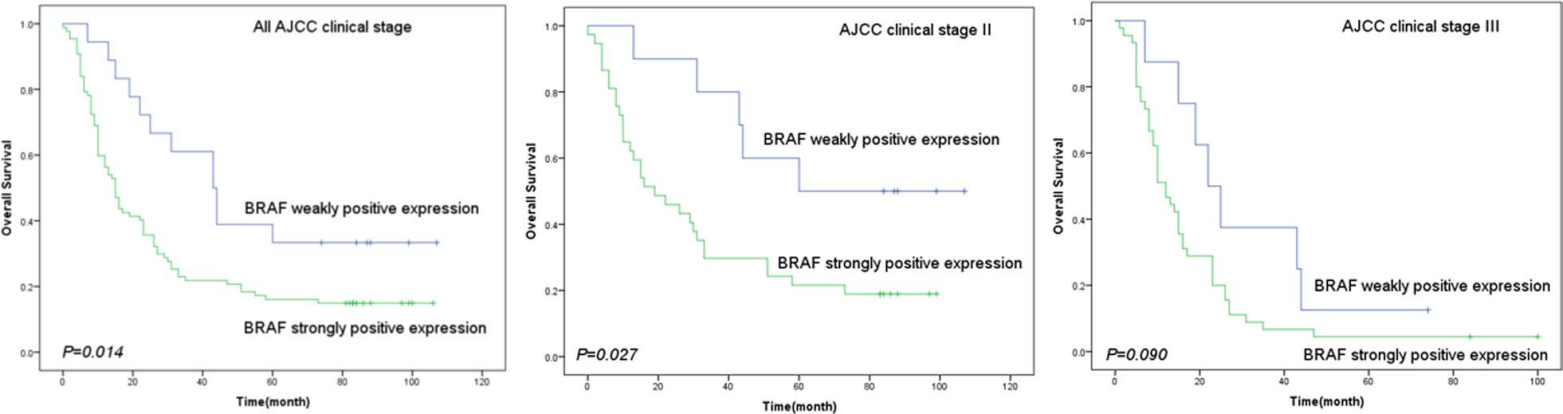
Kaplan–Meier curve analysis for the overall survival (OS) of all AJCC clinical stage, AJCC clinical stage II and AJCC clinical stage III patients according to BRAF expression

To examine the potential role of BRAF expression in EC, we analyzed the transcriptome data of EC patients extracted from the TCGA database. The expression levels of BRAF was significantly increased in EC patients compared to controls (Fig. 2). At the end of the 0–107-month follow-up period, 86 (81.9%) patients died while the remaining 19 (18.1%) patients were still alive. Using the Kaplan–Meier method and log-rank test to analyze the OS of the 105 patients, we found that patients with BRAF strongly positive expression tumors had lower OS compared to patients with BRAF weakly positive expression tumors (*P* = 0.014) (Fig. 3). We further analyzed the respective OS of patients with different AJCC clinical stage. BRAF strongly positive expression showed a significantly shorter OS (*P* = 0.027) in the AJCC clinical stage II subgroup of patients, whereas BRAF strongly positive expression in patients with AJCC clinical stage III did not show a significant correlation with OS (*P* > 0.090) (Fig. 3). We utilized both univariate and multivariate Cox hazard regression models to evaluate every risk factor for OS (Table 3). In the univariate regression analysis, gender [hazard ratio (*HR*) = 1.739, 95% confidence interval (*CI*) = 1.032–2.931, *P* = 0.038], T stage (*HR* = 5.526, 95% *CI* = 1.353–22.574, *P* = 0.017), N stage (*HR* = 1.850, 95% *CI* = 1.192–2.870, *P* = 0.006), AJCC clinical staging (*HR* = 9.119, 95% *CI* = 1.265–65.707, *P* = 0.028) and BRAF strongly positive expression (*HR* = 2.087, 95% *CI* = 1.131–3.853, *P* = 0.019) were poor factors. In the multivariate regression analysis, gender (*HR* = 1.884, 95% *CI* = 1.039–3.133, *P* = 0.036) and BRAF strongly positive expression (*HR* = 2.782, 95% *CI* = 1.444–5.361, *P* = 0.002) were significant prognostic factors of poor outcomes.

**Table 3:** Univariate and multivariate Cox regression analyses of risk factors for esophageal cancer

Variable	Univariate analysis			Multivariate analysis		
	HR	95%CI	*P* value	HR	95% CI	*P* value
Age (60 y vs. > 60 y)	0.949	0.604–1.492	0.820			
Gender	1.739	1.032–2.931	0.038	1.884	1.039–3.133	0.036
T stage (T1 vs. T2–4)	5.526	1.353–22.574	0.017	1.137	0.149–8.949	0.903
N stage (N0 vs. N1–3)	1.850	1.192–2.870	0.006	1.374	0.868–2.176	0.176
AJCC clinical staging (I vs. II–III)	9.119	1.265–65.707	0.028	7.383	0.417–134.348	0.172
Pathological grading (I vs. II–III)	1.234	0.752–2.024	0.405			
Ki67 (< 14% vs. ≥ 14%)	1.539	0.893–2.653	0.120			
BRAF (weakly vs. strongly)	2.087	1.131–3.853	0.019	2.782	1.444–5.361	0.002

### Effect of small interfering RNA (siRNA) knockdown of BRAF in EC cells on cell clone formation

**Fig. 4:**
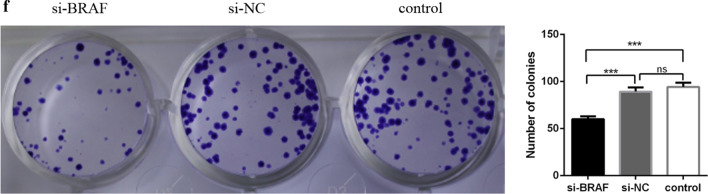
Effect of BRAF on the clonal formation of esophageal cancer cells; ***P < 0.0001

In order to further observe the effect of BRAF on the proliferation of TE-1 cells, we conducted a plate clone formation experiment. In TE-1 cell lines, siRNA knockdown of BRAF (si-BRAF) significantly reduced the cell clone formation rate compared to the si-NC and control group (Fig. 4).

### Effect of siRNA knockdown of BRAF on the migration, invasion, and wound healing of EC cells

**Fig. 5:**
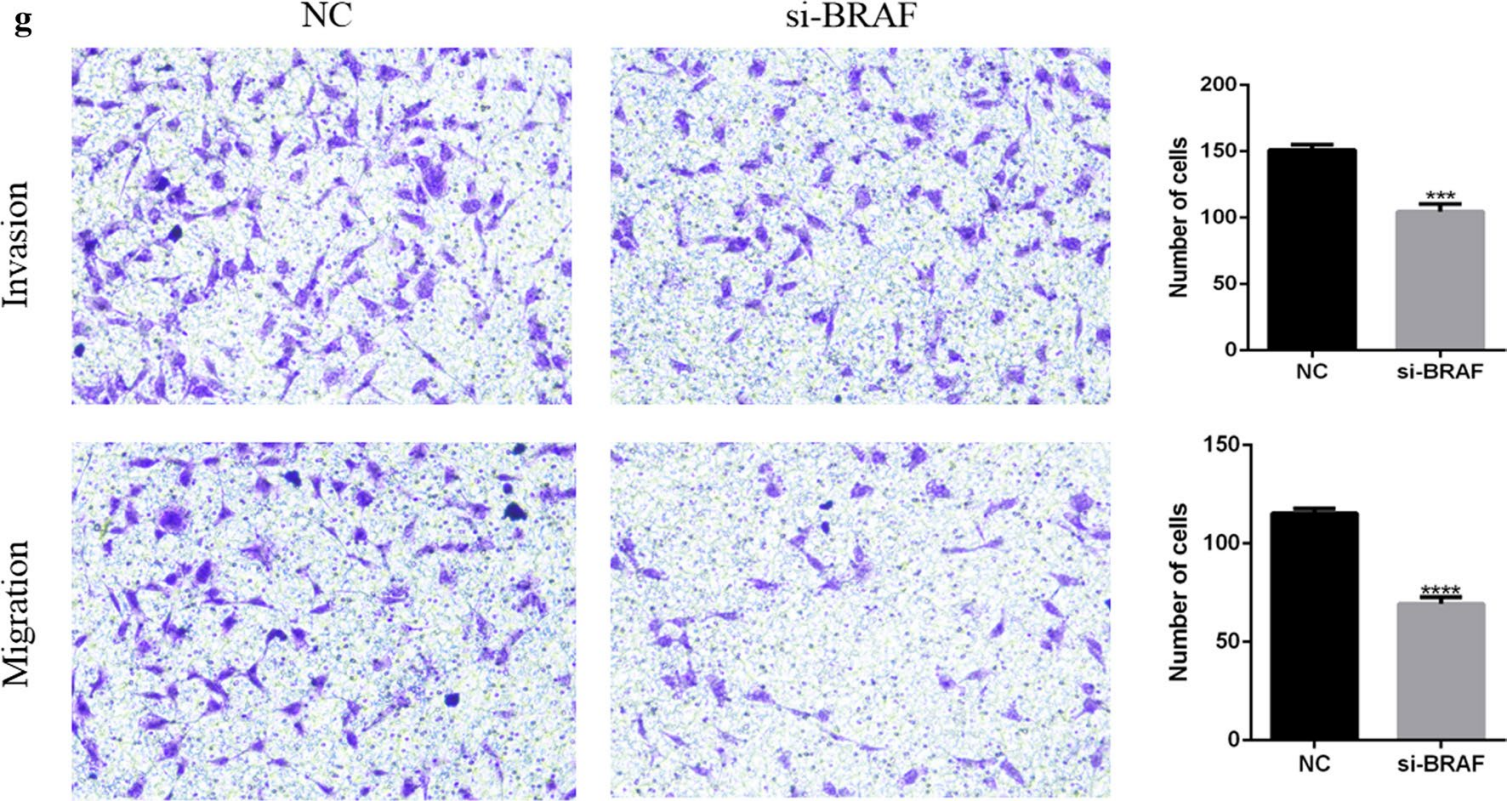
Effect comparison of the siRNA knockdown of BRAF on cell invasion and migration; ***P < 0.0001 and ****P < 0.00001

**Fig. 6:**
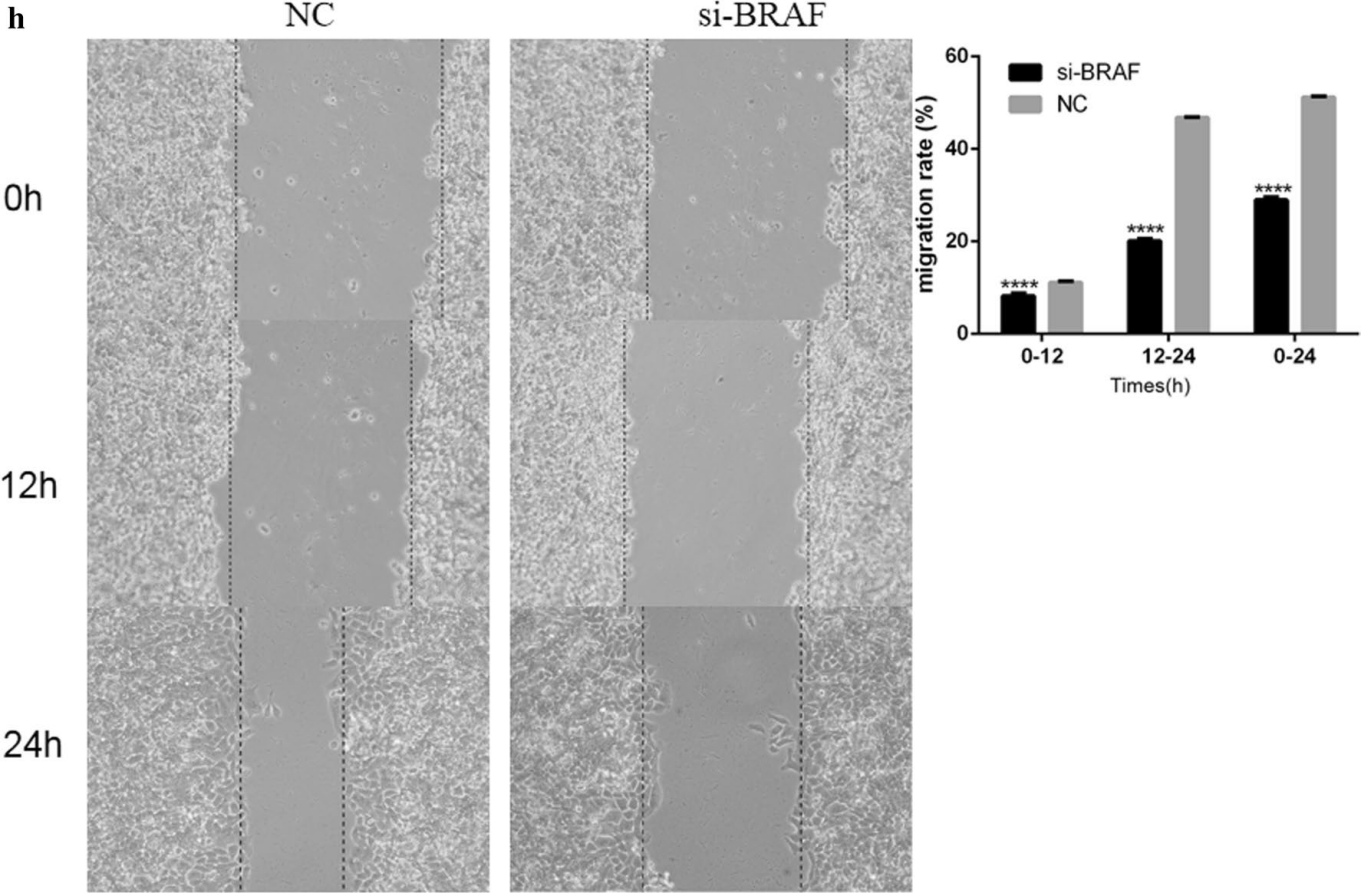
Effect of BRAF knockdown on the wound healing of esophageal cancer cells; ****P < 0.00001

In order to investigate the effect of BRAF on the invasion and migration of TE-1 cells, Transwell chamber and wound healing assays were used. We investigated the effects of BRAF knockdown by si-BRAF on the migration and invasion of TE-1 cells. The results of the Transwell assay showed that the migration and invasion of cells in the experimental group were significantly inhibited compared to the control group, and the inhibition rates of the siRNA group were 67% and 60%, respectively (Fig. 5). In the scratch test, the wound healing ability of the BRAF knockdown group was significantly weaker than that of the control group (Fig. 6). All experimental results were obtained from three independent experimental data.

### Effect of the growth of BRAF inhibitor cell lines on tumor formation in nude mice

**Fig. 7:**
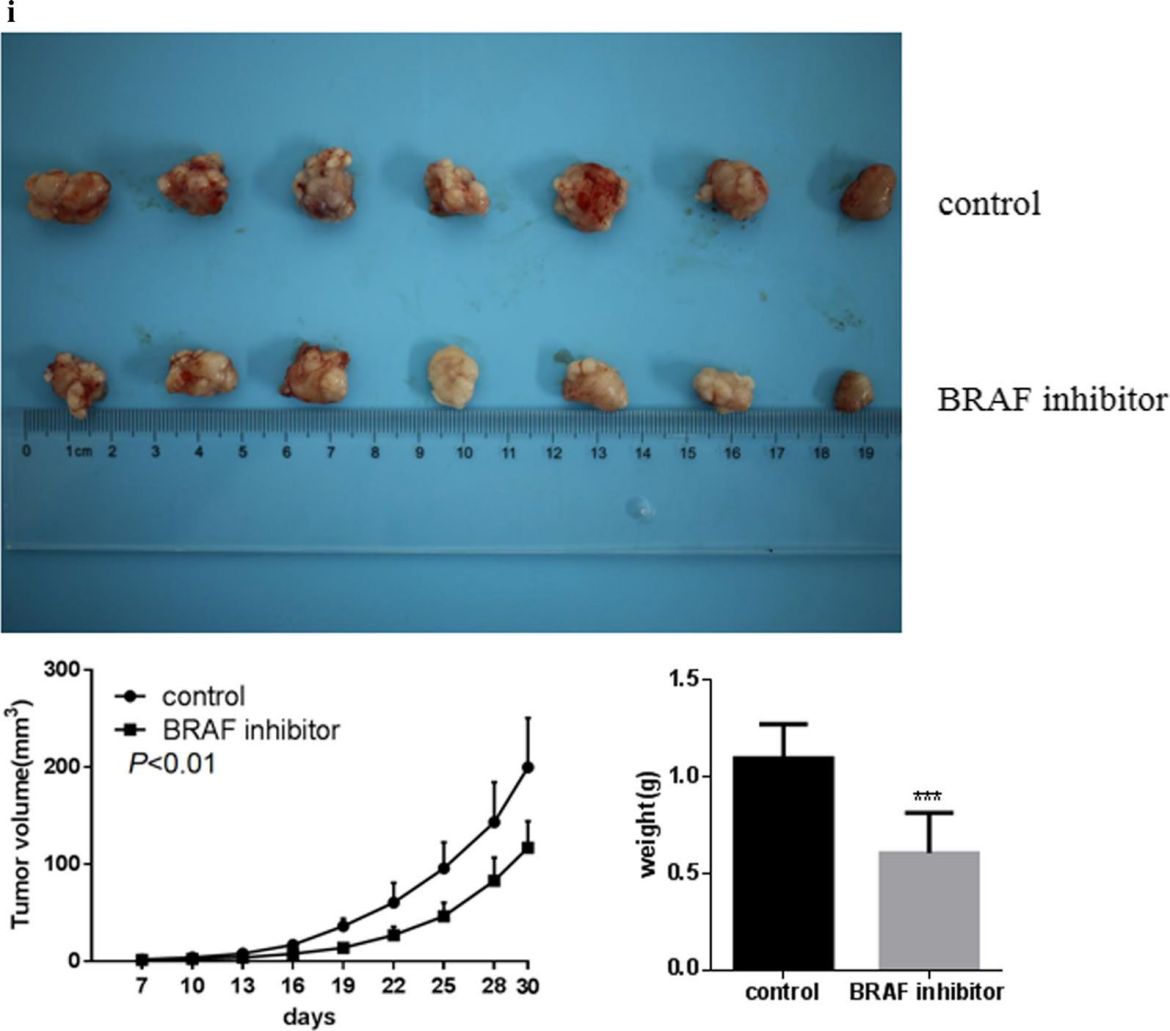
Growth of BRAF inhibitor cell lines in nude mice; ***P < 0.0001

All mice injected with EC cells developed solid tumors in 1 week as revealed by macroscopic examination, the growth rate of tumors in the experimental group was significantly slower than that in the control group, and the size and weight of the tumors excavated after the death of nude mice were significantly smaller than those of the control group. There were significant differences in tumor growth volume and weight between the two groups (Fig. 7).

## Discussion

Individualized treatment and molecular-targeted therapy based on molecular level have been paid increasing attention in recent years. At present, the most novel process of cancer treatment is to detect the gene expression levels and mutations of patients, develop targeted drugs for different gene mutations, and create individualized treatments. The BRAF gene is a kind of oncogene that has been found to play an important role in the occurrence, development, and metastasis of tumors [5]. BRAF gene mutations exist in papillary thyroid cancer, malignant melanoma and colorectal cancer [7–9]. The BRAF gene contains 19 exons, of which exons 11 to 15 encode the kinase region. BRAF gene mutations have been found to occur in exons 11 and 15 with the majority in exon 15 (89%). Approximately 92% of BRAF gene mutations are located in nucleotide 1799 (t → a), resulting in the substitution of valine with glutamic acid (V600E) [10]. BRAF gene mutations significantly increase its kinase activity and continuously activates the mitogen-activated protein kinase (MAPK) pathway, which leads to the phosphorylation of nuclear transcription factors and various proteins and ultimately results in the abnormal proliferation, differentiation, and apoptosis of cells, thereby forming tumors [11].

In this study, we found EC tumor tissue samples 82.9% showed strongly positive BRAF expression and adjacent normal tissues10.7% showed strongly positive expression, which was similar to that of thyroid and colorectal cancers [12, 13]. The results showed that BRAF was expressed at a high frequency in EC tissues. Therefore, BRAF protein can be considered as an oncogene expression product in tumors. The detection of BRAF expression in tissue samples is helpful for the pathological diagnosis of EC.

In this study, we found that BRAF protein overexpression was not related to gender, age, T stage, N stage, AJCC clinical stage, pathological type, pathological grading, and p53 expression, which suggested that BRAF may have universal significance in the occurrence and development of EC. BRAF protein overexpression was related to the survival time of EC patients well as Ki67 expression, indicating that BRAF was a prognostic biomarker of poor outcomes in EC patients with influence on OS. The results were consistent with several previous studies, including one that determined BRAF can be used as a prognostic indicator of colorectal cancer. The higher the positive expression rate of BRAF, the lower the survival rate later tumor staging of patients [14]. Jacobsen et al. [15] showed that the Ki67 expression is related to the survival and prognosis of patients with EC and that patients with high expression of Ki67 have a shorter survival period and poor prognosis. Xu et al. [16] found that p53 and Ki67 protein accumulation can be used as biomarkers for early diagnosis of EC in high-risk populations.

In our study, univariate and multivariate regression analyses showed that BRAF overexpression was a risk factor for poor prognosis of EC. Thus, consistent with the results of previous studies, BRAF may be used as a marker of poor prognosis in EC patients.

In vitro experiments showed that after BRAF knockdown, significantly reduced the cell clone formation rate compared to the control group. The invasion and migration ability of TE-1 cells were inhibited by 67% and 60%, respectively, and the scratch healing ability was decreased by 56%. It has previously been observed that BRAF plays a key role in TE-1 cells, and this section of the experimental results are in line with existing literature reports [17]. In the nude mice tumor-bearing model, inhibition of BRAF activity decreases tumor growth, which indicated that BRAF played a key role in tumor proliferation. After BRAF activation inhibition, cell proliferation was blocked in the G1 phase [18], thus affecting the cancer cell cycle. This observation suggests that BRAF may be a new therapeutic target for EC.

The best-studied BRAF mutations occur at position V600 (V600E and V600K), resulting in constitutive activation of BRAF and downstream activation of MAPK kinase (MEK) and extracellular signal-regulated kinase. The overall mutation rate of BRAF in malignant tumors is 7% but varies with tumor type, and mutations are observed in approximately 50% of patients with melanoma, approximately 25% of patients with anaplastic thyroid cancer, and 2–8% of patients with non-small cell lung cancer [19, 20]. BRAF kinase inhibitor can significantly inhibit the proliferation of melanoma cells with BRAF gene V600E mutations in the G1 phase but has no inhibitory effect on wild-type BRAF [18, 19]. Recent clinical studies have shown that BRAF kinase inhibitors, such as acetinib and plx4032, have been used in the treatment of malignant melanoma [21]. A recent pooled analysis of patients treated with dabrafenib plus trametinib in COMBI-d/v (n = 563) suggested potential stabilization of survival curves with 4- and 5-year progression-free survival (PFS) rates of 21% and 19% as well as 4- and 5-year OS rates of 37% and 34%, respectively [22]. A clinical trial called COLUMBUS has shown that the combination of encorafenib and binimetinib therapy for melanoma has the longest median PFS of 14.9 months and a median overall survival of 33.6 months compared to other BRAF-MEK combination therapies with favorable adverse events [23]. Therefore, BRAF kinase inhibitor can be introduced in the treatment of EC. Therefore, BRAF kinase inhibitors can be introduced into the treatment of EC. By applying BRAF kinase inhibitors to EC cell lines with BRAF gene mutation, we can detect whether the expression of differentiation markers is decreased, whether the tumor cells have proliferation arrest, whether the activity of BRAF protein kinase is down regulated, and whether the relative expression of nuclear related genes is decreased, To determine whether the invasion and metastasis of the cells are reduced, so as to obtain a more accurate relationship between BRAF mutation and the occurrence and development of EC. Exploring the basic research theory of BRAF inhibitors in the clinical treatment of EC will help to provide a new method for comprehensive EC treatment.

## Conclusion

In conclusion, our results suggested that BRAF overexpression correlated with lower overall survival in esophageal cancer patients. Thus, BRAF may serve as a valuable predictive biomarker and therapeutic target for EC.

## Data Availability

The datasets generated and analyzed during the current study are available from the corresponding author on reasonable request.

## Abbreviations

BRAF: V-Raf murine sarcoma viral oncogene homolog B1
EC: Esophageal cancer
EGFR: Epidermal growth factor receptor
KRAS: Kirsten ras 1
T stage: Tumor infiltration
N stage: Lymph node metastasis
AJCC: American Joint Committee on Cancer
TMN: International Union Against Cancer tumor, node, metastasis classification system
IHC: Immunohistochemistry
FBS: Fetal bovine serum
OS: Overall survival
si-BRAF: SiRNA knockdown of BRAF
NC: Normal environment control group
MAPK: Mitogen-activated protein kinase
MEK: MAPK kinase
TE-1: Human esophageal cancer cell
